# 3-D real-time ultrasound tracking of acoustically actuated swimming microdrone

**DOI:** 10.1038/s41598-024-52044-0

**Published:** 2024-01-18

**Authors:** Qiyang Chen, Fang-Wei Liu, Sung Kwon Cho, Kang Kim

**Affiliations:** 1 Department of Medicine, University of Pittsburgh, 623A Scaife Hall, 3550 Terrace Street, Pittsburgh, PA 15261 USA; 2 Center for Ultrasound Molecular Imaging and Therapeutics, Department of Medicine & Heart and Vascular Institute, University of Pittsburgh School of Medicine and University of Pittsburgh Medical Center (UPMC), Pittsburgh, PA 15261 USA; 3 Department of Mechanical Engineering and Materials Science, University of Pittsburgh, Pittsburgh, PA 15261 USA; 4 Department of Bioengineering, University of Pittsburgh, Pittsburgh, PA 15261 USA; 5 Division of Cardiology, Department of Medicine, University of Pittsburgh School of Medicine, Pittsburgh, PA 15261 USA; 6 McGowan Institute of Regenerative Medicine, University of Pittsburgh and University of Pittsburgh Medical Center (UPMC), Pittsburgh, PA 15219 USA

**Keywords:** Biomedical engineering, Engineering, Mechanical engineering

## Abstract

Maneuverable microswimmers/microdrones that navigate in hard-to-reach spaces inside human bodies hold a great potential for various biomedical applications. Acoustically actuated microswimmers have already demonstrated feasibility. However, for eventual translation of this technology, a robust 3-D tracking strategy for the microswimmer is particularly required. This paper presents our lab-designed 3-D ultrasound tracking system for real-time tracking of an acoustically actuated 3-D swimming microdrone. The ultrasound tracking system utilizing two ultrasound probes, a step motor and a host controller, was built to track the 3-D arbitrary motion of the microdrone in real-time. The performance of tracking was evaluated in the benchtop experiments by comparing the reconstructed trajectories with synchronized camera recordings. The ultrasound tracking system showed high reliability, with an average error of less than 0.3 mm across six different trials when compared to camera tracking. The results demonstrated the capability of our lab-designed 3-D ultrasound tracking system in accurately tracking the undetermined motion of the acoustic actuated 3-D swimming microdrone in real-time. The developed tracking system holds promise as a potential approach for biomedical applications and could pave the way for future clinical translation of the microswimmer technology.

## Introduction

Maneuverable microswimmers/microrobots/microdrones that can be propelled to navigate in hard-to-reach spaces and microfluidic environments inside human bodies hold a great potential for serving as a navigating cargo for various biomedical applications, including targeted drug delivery, biosensing, minimally invasive surgery, tissue regeneration^1–6^. However, two main challenges must be overcome before practical in vivo applications can be realized: remote propulsion and tracking strategies. Several propulsion principles have been explored to drive and manipulate microswimmers, such as self-propulsion by chemical fuels^7–9^, external forces by light^10^, magnetic^11–13^ or acoustic fields^14–18^, and hybrid actuation^19,20^.

Among these, acoustic actuation holds several advantages, including noninvasiveness, nontoxicity to organisms, deep tissue penetration, and economic in translations. One of the mechanisms in acoustic propulsion is based on the fluid mechanical fact that if the Reynolds number is not too small, the fluid intake and discharge from the small tube is asymmetric^21^. Utilizing this mechanism, an acoustically actuated bubble-powered microswimmer has been developed using microfabrication technology, demonstrating swimming motions under acoustic waves^22,23^. In this design, a gaseous bubble is trapped in the cylindrical tube fabricated on the microswimmer when immersed in water due to the hydrophobicity of the tube interior. Acoustic wave induces periodical oscillation of a gaseous bubble and the back-and-forth motion of the gas–liquid interface results in a non-zero time-averaged flow field around the outlet of the tube. Therefore, the asymmetric flow creates an outgoing flow that propels the main body of the microswimmer opposite to the opening of microtubes. Microswimmers that are capable of steering in two-dimensional planes by selectively activating the microtubes with varying lengths and orientations have been designed^17,24^. Moving forward, attempts have been made to further implement the microswimmer propulsion in 3-D using different strategies, for example, adding acoustic radiation force^25^ or magnetic force^26^ to provide the propulsion in the third direction. Our group has designed a 3-D swimming microdrone solely directed and powered by the acoustically actuated microstreaming and shown its capabilities navigating in 3-D space in vitro^18,27^.

Although the 3-D maneuverable microswimmer has been proposed, their tracking and control remain a challenge for eventual translation of the technology into practical in vivo applications. A robust tracking strategy is indispensable to locate the microswimmer during manipulation for biomedical tasks and provide real-time feedback to the control system to monitor and correct its trajectory. However, developments of tracking strategies that could be appropriate for in vivo applications have rarely been made. Among the past developments that capture the 2-D microswimmer behaviors^17,28^, as well as further implement the 3-D tracking^28–30^, visual cameras were mostly used to record the microswimmer, which are not an applicable solution in vivo mainly due to the limited optical imaging depth in tissues. Ultrasound tracking offers a larger imaging depth, reasonable spatial and temporal resolution, compatibility with the actuation mechanism, safety, and low cost, making it a suitable candidate for tracking microswimmers in biomedical applications. Various ultrasound localization and tracking strategies have been demonstrated for micro-robots, primarily for those magnetically actuated. These include brightness-based strategies operating on B-mode images utilizing contrast differentiation^31^, template matching^32,33^, and deep learning^34^, as well as motion-based techniques employing Doppler^35,36^ or Acoustic Phase Analysis^37,38^. In the earlier studies by our group, the ultrasound tracking using brightness-based strategy of the acoustically activated 2-D microswimmer was validated through in vitro experiments in which the accuracy and reliability of ultrasound tracking for various 2-D trajectories were demonstrated^39^. Considering the future practical applications in vivo that the microswimmer travels in 3-D space, a system that can follow and track the microswimmer with undetermined trajectories in 3-D in a reliable and real-time manner, is particularly required and largely unaddressed.

In this paper, we present a lab-designed 3-D ultrasound tracking system that can track the motion of the 3-D swimming microdrone in a water tank in real-time. The tracking performance was evaluated in an in vitro experimental set-up by comparing the reconstructed trajectories of a total of six trails with a synchronized camera recording.

## Methods

### Design of the 3-D swimming microdrone

**Figure 1:**
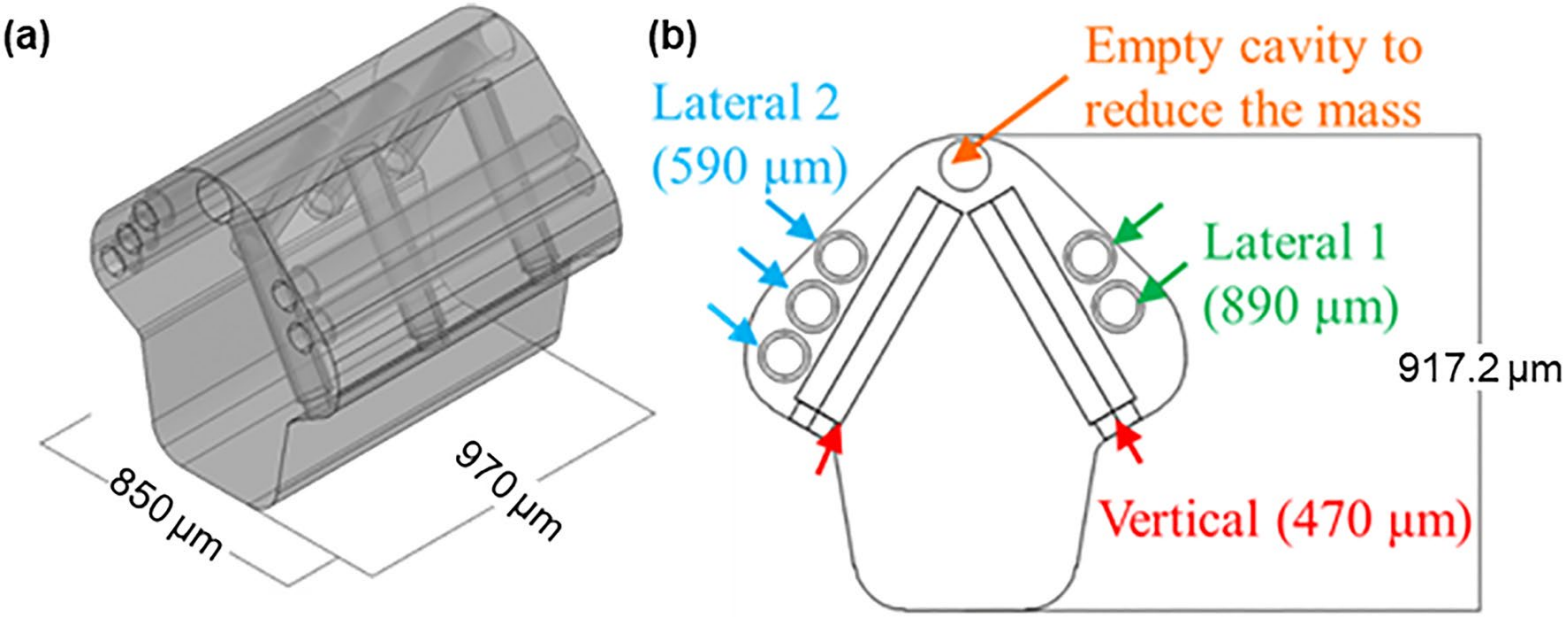
The design of 3-D micro swimmer drone. (**a**) Overall view; (**b**) front view.

Figure 1 shows the design of the 3-D swimming microdrone that is capable of multi-directional movement^18,27^. The microdrone is equipped with three types of microtubes in the diameter of 100 μm, namely “Lateral 1” (890 μm long × 2), “Lateral 2” (590 μm long × 3), and “Vertical” (470 μm long × 6), which are strategically positioned and oriented throughout the body of the drone to enable navigations in three dimensions. The interior surface of each microtube is a hydrophobic that automatically traps an air bubble when the dry tube submerges in water. Under the activation of acoustic waves, the air bubbles oscillate to produce propulsion forces in directions opposite to the opening of the microtubes. The literature outlines the propulsion force generated by bubble oscillation using the formula, $F={0.8\rho A(af)}^{2}$, in which F is the propulsion force, A is the cross-sectional area of the tube opening, $\rho$ is the density of fluid, f and a represent oscillating frequency and amplitude^17^. The propulsion force maximizes as the oscillating frequency reaches the resonant frequency, which is related to the length of the microtube as shown in the formular ${f}_{0}=\frac{1}{2\pi }\sqrt{\frac{\kappa {P}_{0}}{\rho {L}_{0}{L}_{B}}}$, where ${L}_{B}$ represents the length of the bubble and ${L}_{0}$ is the length of water column between the bubble interface and the tube opening^21^. The resonant frequencies of these three types of microtubes are 5.9 kHz, 7.9 kHz, and 11.7 kHz, respectively based on the design. The 3-D propulsion can be achieved by individually or simultaneously actuating the microtubes: (1) propelling upward by Vertical, (2) yawing clockwise or counterclockwise by Lateral 1 or 2 respectively, (3) moving forward by Lateral 1 and 2 simultaneously at 6.3 kHz and (4) downward by gravity. A dummy cavity with both ends open, positioned in the top corner of the microdrone, is included to reduce the weight in the upper part of the swimmer such that the swimmer has tendency in maintaining upright posture. For more details, refer to^18,27^.

### 3-D ultrasound tracking system and in vitro experimental set-up

**Figure 2:**
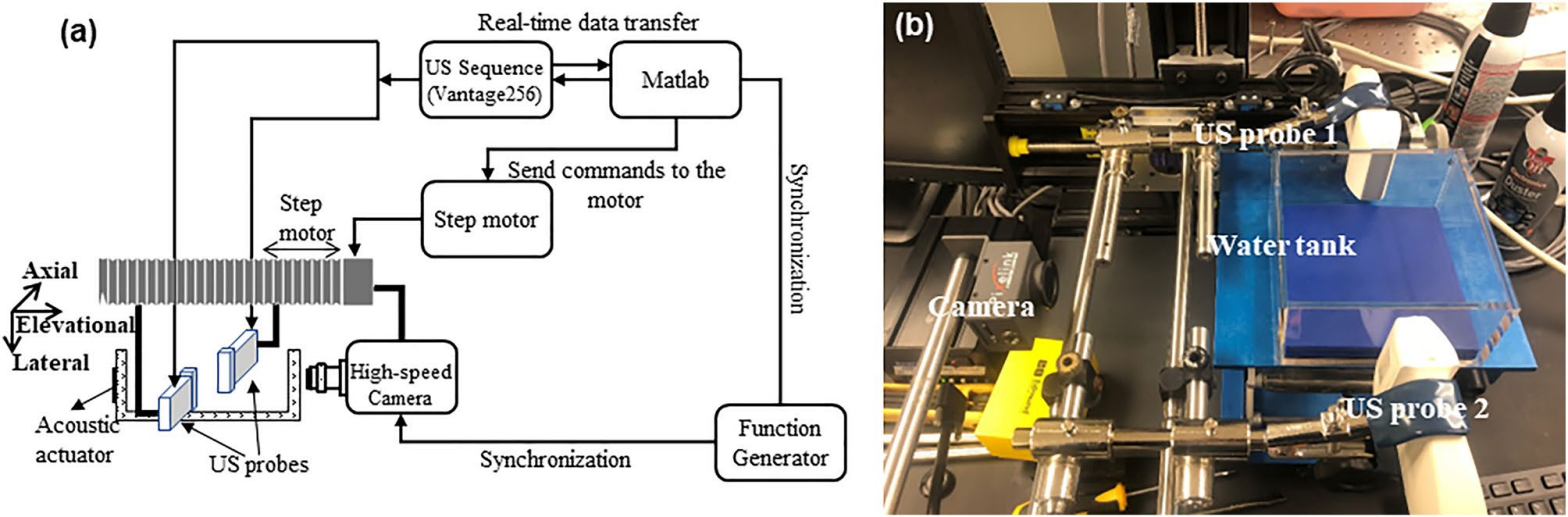
(**a**) Schematic and (**b**) photograph of the experimental setup.

Figure 2 depicts the schematic of the 3-D ultrasound tracking system (a) and a photograph of the in vitro experimental set-up (b). The 3-D microdrone is submerged in a water-glycerin solution (10:1) in the water tank and activated by an acoustic piezo actuator glued to the tank outer surface. The solution has the density close to the 3-D microdrone, thus creating neutral buoyancy to facilitate its actuation in the vertical direction. The piezo actuator to drive the microswimmer in a three-dimensional trajectory is excited by a continuous waveform electrical signal of 10.7 kHz and 99 Vpp generated and amplified by a function generator (Agilent 33250A) and an amplifier (Trek PZD700A). The actual trajectory may differ among trials mainly due to non-unifrom media friction.

**Figure 3:**
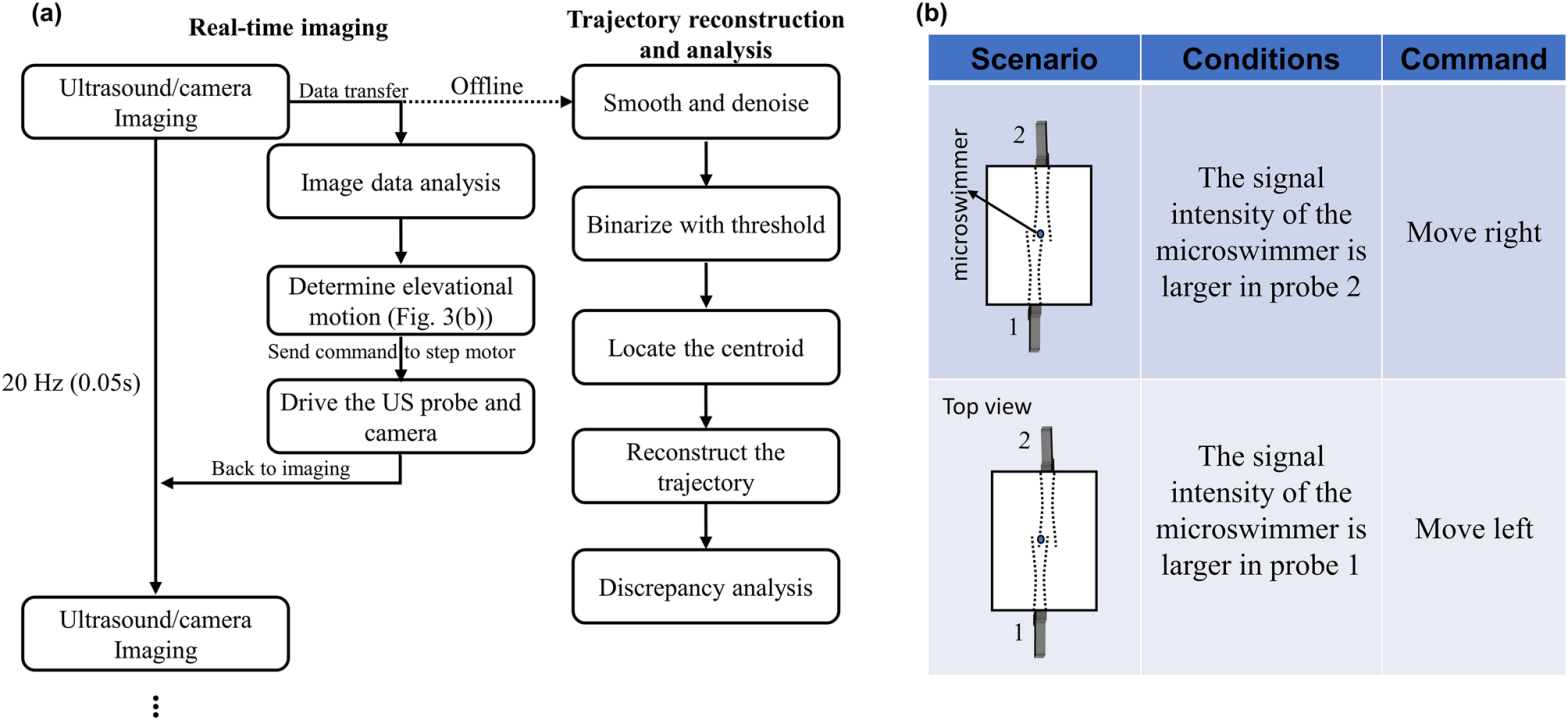
(**a**) Block diagram of ultrasound tracking and trajectory reconstruction algorithm. (**b**) Two scenarios for step motor control.

The 3-D tracking system is configured by two ultrasound probes, a step motor, and a host controller to perform the real-time tracking of the arbitrary motion of the swimming microdrone. Two ultrasound probes (L7-4, ATL) are positioned facing each other with some overlap in their elevational beams and are operated by a programmable ultrasound system (Vantage256, Verasonics, Redmond, WA) to identify the microswimmer's motion. The ultrasound probe operates at a center frequency of 5 MHz (60% bandwidth) and provides a field of view of 38 mm in lateral and about 50 mm in axial at the spatial resolution of 0.3 mm and 0.148 mm, respectively. Ultrasound plane wave imaging (1 angle at 0°, 5.2 MHz, 1 cycle per pulse) was used to acquire the microswimmer images. At the beginning of each experiment trial, the microswimmer is placed in the overlaps of the elevation beams of the two probes to assure it to be seen by both probes. Any motion in axial and lateral directions can be tracked in the field of view of the two probes from their initial positions. The ultrasound images from the two probes are transferred and analyzed in real-time by the motor control algorithm, which senses the microswimmer’s elevational motion and sends traversing commands to the step motor to drive the probes along the elevational. The host controller was configured in the tracking system to receive and analyze the ultrasound acquisitions and send commands to the step motors in real-time. The frame rate of ultrasound tracking is 20 Hz. The process between two image acquisitions, including image data transfer, image analysis, and motor motion, is completed within 0.05 s to ensure real-time implementation. In this way, the microswimmer moving in an arbitrary trajectory can be kept within the ultrasound field of view and captured at all time. The real-time operating sequence is illustrated in Fig. 3a.

The tracking accuracy is evaluated using a high-speed camera (PL-D732CU-T, Pixelink, Rochester, NY), which is held on the step motor and operated at the same frame rate of ultrasound. The field of view of the camera measures about 50 mm in axial (z axis) and 28 mm in lateral (y direction) with a spatial resolution of 0.026 mm. The imaging field of view (focal plane) is maintained within the overlapping ultrasound imaging planes and adjusted along the elevational axis with the same traversing commands. The tracking system is synchronized by an external trigger from a function generator and implemented in Matlab (MathWorks, Natick, MA). The reconstructed trajectories from both ultrasound and camera acquisitions are compared offline to evaluate the tracking accuracy.

### Real-time image sequence

Figure 3a illustrates the block diagram of the real-time tracking sequence and the offline trajectory reconstruction algorithm. The ultrasound and the synchronized camera are operated at the frame rate of 20 Hz. In the interim between image acquisition events, the ultrasound imaging data is promptly transmitted to the host computer for real-time processing, specifically for elevational tracking. The control algorithm undertakes an analysis of the image data to determine the microdrone's elevational motion direction. Subsequently, commands are relayed to drive the probes and camera, accordingly, as described previously. A motor control strategy is designed to ensure the microdrone's consistent presence within the ultrasound field of view throughout the tracking process. The control algorithm leverages a specific ultrasound image feature within the elevational beam (slice thickness) of the ultrasound probe, where an object positioned closer to the beam's center line displays stronger contrast^40^. To exploit this, we intentionally offset the placement of the two ultrasound probes. Figure 3b illustrates two scenarios considered in the control algorithm for driving the step motor. By computing intensity ratios between the brightest point and surrounding points across each pair of ultrasound images, we determine the elevational location of the object with respect to the probes. This method allows us to identify the object's position relative to the probes and subsequently adjust the step motor to accurately follow the elevational motions. To ensure that the microswimmer remains within the overlapped beams between two imaging frames all the time, the motor speed is set to 2.5 mm/s, which is sufficient to follow the motion of the microswimmer.

### Analysis of the tracking results

To evaluate the tracking accuracy of the developed 3-D ultrasound tracking strategy, a self-written particle tracking algorithm is applied in Matlab to reconstruct the trajectories from ultrasound and camera acquisitions^39^. The motion of the microswimmer in *y* (lateral) and *z* (axial) coordinates is recorded by both ultrasound and camera. The ultrasound imaging renders the microswimmer as a high-contrast bright spot within the images. As a signal preprocessing step in the tracking algorithm, a median filter is initially applied to all frames to minimize potential salt-and-pepper noise. Subsequently, the video frames undergo thresholding to convert them into binary images. Within these binary images, the microswimmer's surface area is estimated, and its geometrical center is established as the representative position of the microswimmer. In the ultrasound tracking method, the position is determined by averaging the results from the two probes to minimize errors and enhance reliability. The motion in the elevational direction (*x*-coordinate) is roughly obtained by the motor movement, which is recorded throughout the tracking. Therefore, the trajectory in three-dimensional space can be reconstructed by both ultrasound and camera. The ultrasound tracking accuracy is evaluated by comparing the results from 6 experimental trials to those obtained by the camera, which is considered the gold standard in this experiment. The tracking error (*E*) is defined as the discrepancy in the positions between the ultrasound and camera trajectories at each frame. The normalized error, defined as the error (*E*) divided by the total moving distance ($s=\sqrt{\sum_{0}^{t}{(\Delta x)}^{2}+{(\Delta y)}^{2}}$), is also calculated to assess whether the error accumulates.

## Results and discussion

**Figure 4:**
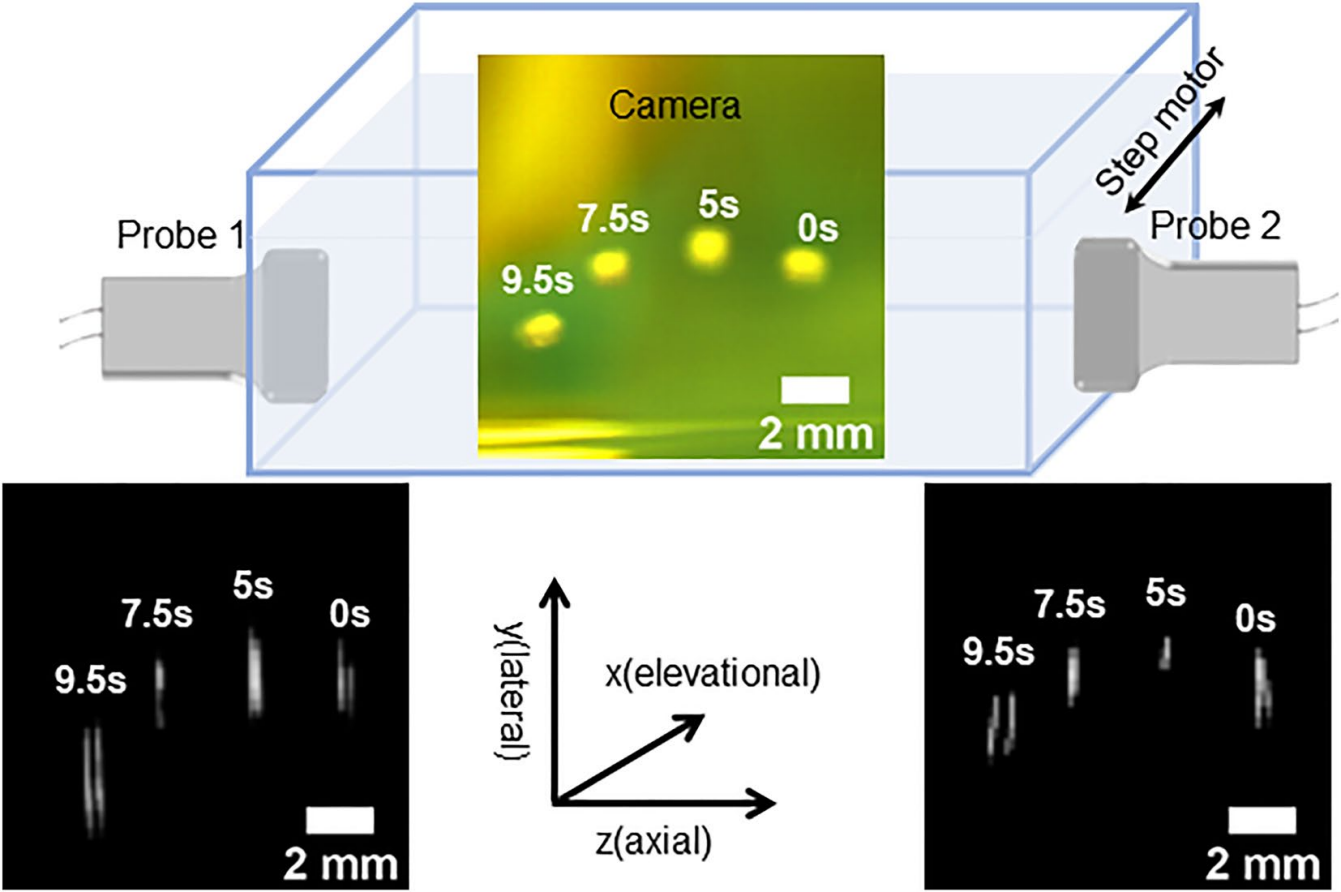
Superimposed time-lapse images from the 3-D motion of the microswimmer acquired by the camera (top) and two ultrasound probes (bottom). The microswimmer motion is shown within the schematic of the experiment set-up along with the 3-D coordinates.

Figure 4 displays the superimposed time-lapse images of the microswimmer captured by camera (top panel) and two ultrasound probes (bottom panel) from a representative trial. The experimental set-up is presented in the schematic diagram, which depicts the water tank, the probe orientation, and the coordinates. The microswimmer motion in the lateral-axial coordinate (*y–z* plane as noted in Fig. 4) can be imaged by ultrasound and camera at the initial position. As the microswimmer moves in the elevational direction (*x* direction), the motor control algorithm analyzes the information from ultrasound and determines the moving direction to drive the motor that holds the two probes and the camera to follow the moving microswimmer. In general, the motion in the *y–z* plane can be captured by both camera and ultrasound, while the *x*-direction motion is roughly obtained by the motion of the motor.

**Figure 5:**
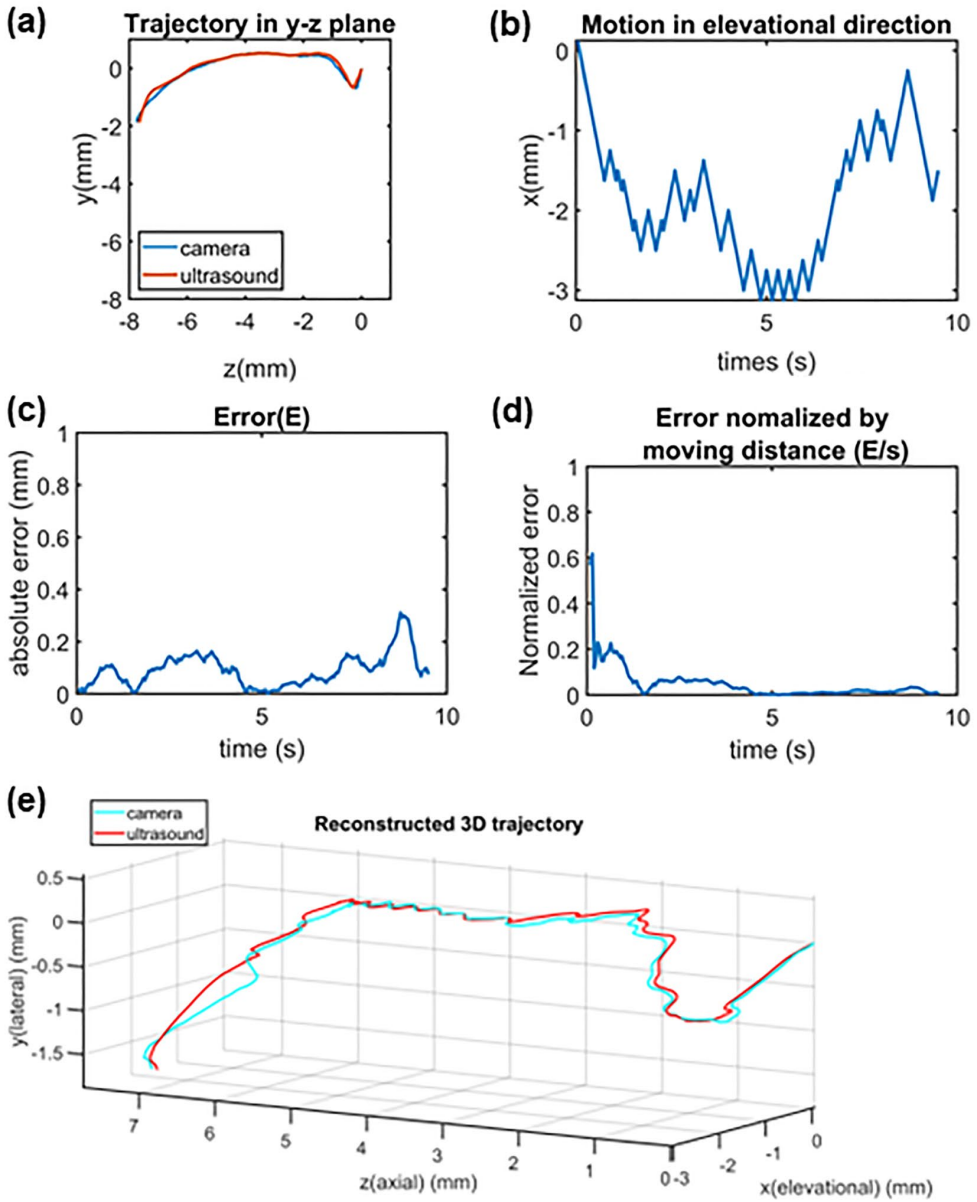
The tracking results from the representative experimental trail. (**a**) Reconstructed trajectories in y–z plane by camera and ultrasound; (**b**) step-motor displacement in elevational direction; (**c**) error (E) of ultrasound tracking compared to camera tracking (discrepancy between ultrasound and camera trajectory) at each frame; (**d**) error (E) of ultrasound tracking normalized to moving distance ($s=\sqrt{\sum_{0}^{t}{(\Delta x)}^{2}+{(\Delta y)}^{2}}$) at each frame. (**e**) Reconstructed 3-D trajectory of the microswimmer by the ultrasound and camera.

Figure 5a shows the reconstructed trajectory in the y–z plane from the camera and ultrasound tracking of the experimental trial in Fig. 4. It is found that the trajectory by ultrasound tracking is overall in good agreement with the trajectory by the camera. Figure 5b shows the step-motor displacement, which roughly corresponds to the elevational motion of the microswimmer. The tracking error (*E*) at each frame, which is defined as the discrepancy in the positions between ultrasound and camera trajectories, is shown in Fig. 5c. The normalized error (*E/s*) at each frame is presented in Fig. 5d. Figure 5e presents the reconstructed 3-D trajectory combining the information of ultrasound and the motor. The movement of the microswimmer recorded by ultrasound and camera, as well as the real-time discrepancy can be found in the supplementary material.

**Figure 6:**
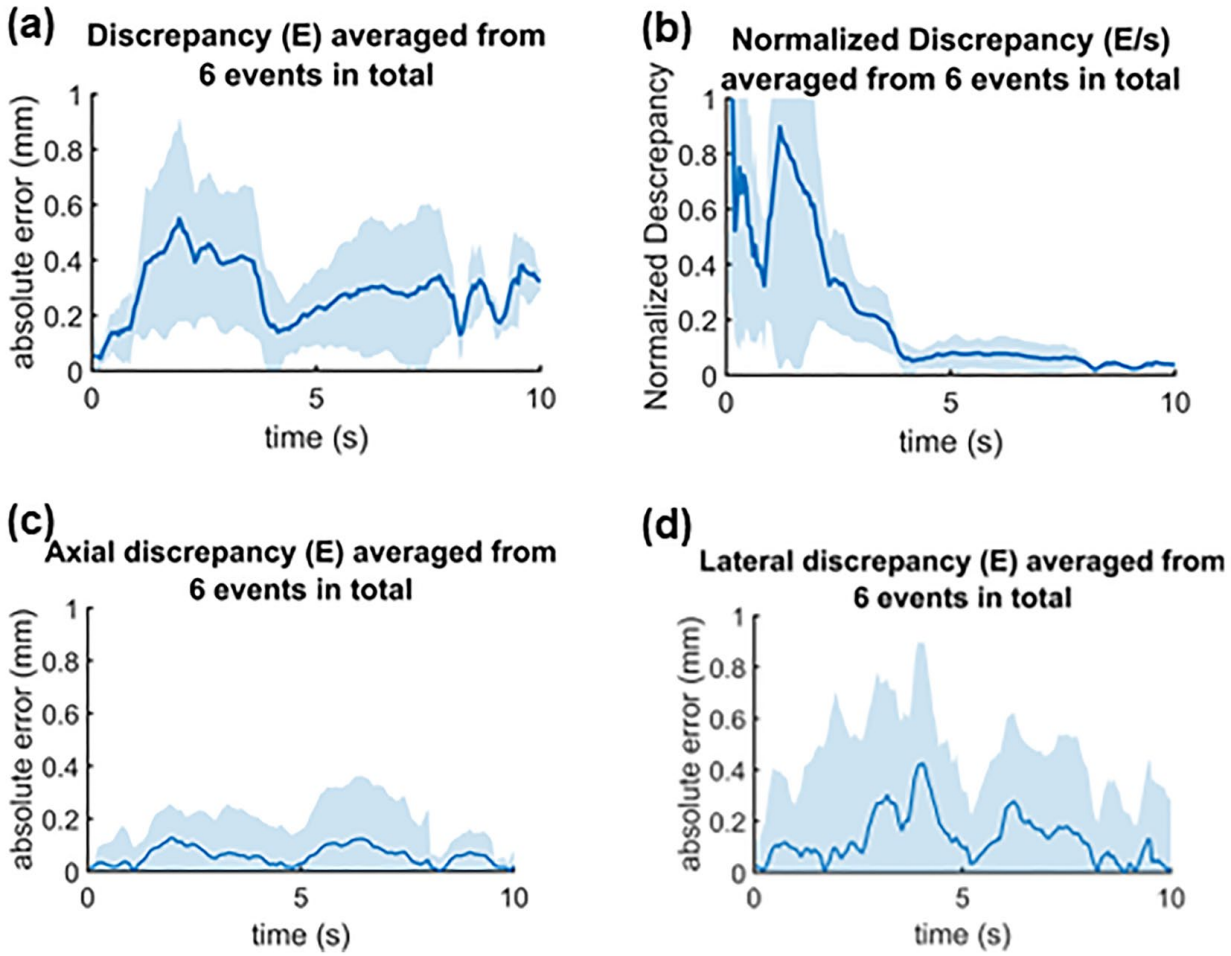
Tracking discrepancy averaged from a total of 6 arbitrary trials. (**a**) The discrepancy between ultrasound and camera tracking at each frame averaged from total of 6 events; (**b**) error (E) normalized to moving distance (s) at each frame averaged from 6 trials; (**c**) The discrepancy in axial axis averaged from a total of 6 events; (**d**) The discrepancy in lateral axis averaged from a total of 6 events.

A total of 6 experimental trials were conducted to evaluate the ultrasound tracking performance. Due to the friction in the fluid, the actual trajectories of the 3-D microswimmer varied across trials. This variation provided independent trials with different and undetermined swimming trajectories to evaluate the designed 3-D ultrasound tracking system. Figure 6 shows the error analysis including the absolute error (*E*) and the normalized error (*E/s*) combining all 6 trials with the shaded error bar indicating the standard deviation. As shown in Fig. 6a, the average tracking errors at each time point along the tracking process remained under 0.6 mm, which is smaller than the size of the microswimmer. The tracking error averaged from all time points is 0.29 ± 0.11 mm, close to the ultrasound lateral resolution. The mean normalized error kept decreasing as the microswimmer runs further, which indicates no accumulation of system errors as the microswimmer travels. It therefore can be expected that when the microswimmer travels a quite long distance, the error of this ultrasound tracking approach stays low over the entire course of travel. It should be noted that there is no feedback control involved for the propulsion of the microswimmer. Therefore, this tracking system demonstrates its real-time ability to track the undetermined motion in the three-dimensional space. As an indispensable component, the demonstrated tracking reliability of real-time availability would readily facilitate the real-time feedback control of the microswimmer manipulation. Overall, the results demonstrate the capability of the tracking system to follow the microswimmer motion in three dimensions and provide reliable tracking inputs for real-time feedback control.

The tracking error is mainly attributed to the relatively low spatial resolution along the lateral direction of the ultrasound probe compared to the camera. The tracking error averaged over all 6 trials in axial and lateral directions are shown in Fig. 6c and d, respectively. A larger error was observed in the lateral direction. The ultrasound lateral resolution (*y* coordinate) of the probes, which is determined by the pitch of the piezo-ceramic elements of the probe, is approximately 0.3 mm, much lower than ultrasound wave resolution in axial (*z* coordinate) direction (~ 0.148 mm) and the camera resolution (~ 0.026 mm). In general, the errors observed in ultrasound tracking mostly fall within a reasonable range, reflecting the spatial resolution of the probe. Importantly, these errors do not exhibit accumulation, indicating no systemic errors attributable to the methodologies employed. Compared to our previous study of 2-D ultrasound tracking of the 2-D microswimmer^39^ that keeps the error under 0.3 mm, a plane wave ultrasound imaging, instead of focused imaging, was employed in this study to provide a high frame rate for real-time operation, while the imaging spatial resolution was compromised and therefore could have increased the tracking error. Moreover, the additional 3^rd^ dimension and the relatively larger size of the 3-D microdrone may have contributed to the relatively larger tracking errors. Also, note that the sizes of the microdrone in each dimension are different, so the error could become larger as it rotates. The shape of the ultrasound image of the microdrone changes as its orientation to the ultrasound probe changes.

The work presented in this paper is one step advancement from the earlier 2-D ultrasound tracking study by extending the entire tracking system into 3-D and towards the real-time manner. Specifically, the micro-robot utilized in this paper features a novel and sophisticated design that enables the propulsion in 3-D by acoustic waves compared to the previous 2-D design. The ultrasound tracking part has evolved to operate in full 3-D, allowing us to identify and continuously track the micro-drone's arbitrary motions in a 3-D space using clinically available 1D array probes. The system integrates and facilitates the communication between the imaging operation, host computer and the step motor, enabling a real-time automatic translation of the probes along the elevational directions to continuously track the microdrone. With this approach, the elevational motion, which cannot be measured by a conventional clinical imaging probe, can therefore be determined and feedback to the system in a real-time manner. The technical advancement and real-time realization for 3-D tracking from 2-D tracking would set a critical foundation towards the future in vivo applications. Note that as our study's scope primarily focuses on demonstrating the ultrasound tracking. A feedback-controlled manipulation of the microdrone was not included in our system, resulting in the absence of real-time reconstruction of micro-drone trajectory. Nevertheless, leveraging the system's real-time communication framework, which analyzes ultrasound acquisition, displaying the microdrone locations, identifies elevational motions, and controls the step motor, offers a promising pathway for potential real-time reconstruction and feedback of microdrone locations in future iterations.

Although the 3-D tracking system has shown promising, there is still room for further technical enhancement. At some instances, the error-to-microdrone-length ratios appear relatively high. This is mainly due to constraints imposed by the hardware, particularly the limitations in the probe center frequency and footprint. As our study primarily focuses on demonstrating the feasibility of 3-D tracking as a proof-of-concept, these occasional high ratios were anticipated. To further enhance the tracking accuracy in future, especially for specific applications, employing a probe with higher center frequency and finer footprint presents a viable solution, which is readily compatible with our system. Additionally, incorporating a state estimator, such as SCD estimator^41^ or Kalman Filter^42^, could be a potential approach to refine the ultrasound tracking outcomes. The state estimator corrects the ultrasound tracking results by using predictions from a dynamic model of the microswimmer so that the accuracy of the ultrasound tracking can be improved to match the true trajectory. The current imaging frame rate of 20 Hz is determined by the signal transfer and processing speed of the computer to ensure a real-time implementation. It can be further enhanced for tracking an even faster motion as a powerful host computer is used.

Moving forward, further evaluation of the 3-D ultrasound tracking system is necessary to enhance its potential for clinical translations. Specifically, testing the accuracy of the 3-D tracking system in ex-vivo animal tissues, such as porcine eyeball cavity, or vessel mimicking phantom, would provide valuable insights for the practicality of this technology. This would serve as a preliminary test for one of the potential applications of the microswimmer that navigates through the eyeball cavity towards the retina to deliver drugs. In the future, a feedback control algorithm for microswimmer propulsion would be added when it is ready to maneuver the motion of the microswimmer in the desired trajectory by adjusting the actuation frequency and amplitude based on the feedback from the ultrasound tracking. Therefore, experiments with more controlled 3-D propulsion and tracking can be envisioned to further refine the translation of the technology.

## Conclusions

In conclusion, our lab-designed 3-D ultrasound tracking system can reliably track the undetermined motion of the acoustically actuated 3-D swimming microdrone in real-time. The reliability of the tracking system was demonstrated with an averaged error of less than 0.3 mm by comparing the results to those obtained from camera tracking throughout six different trials conducted on benchtop. The tracking system can be readily integrated with feedback-controlled propulsion. Providing the advantages of ultrasound in practical applications, our designed tracking system could be a potential approach for tracking the microswimmer motion three dimensionally in biomedical applications and may promote the future clinical translation of the microswimmer technology.

### Supplementary Information

Supplementary Video 1.

## Data Availability

All experimental data are available upon request from the corresponding author.
